# A machine learning-based model analysis for serum markers of liver fibrosis in chronic hepatitis B patients

**DOI:** 10.1038/s41598-024-63095-8

**Published:** 2024-05-27

**Authors:** Congjie Zhang, Zhenyu Shu, Shanshan Chen, Jiaxuan Peng, Yueyue Zhao, Xuan Dai, Jie Li, Xuehan Zou, Jianhua Hu, Haijun Huang

**Affiliations:** 1 Center for Plastic & Reconstructive Surgery, Department of Dermatology, Zhejiang Provincial People’s Hospital (Affiliated People’s Hospital, Hangzhou Medical College), Hangzhou, 310014 Zhejiang China; 2 Center for Rehabilitation Medicine, Department of Radiology, Zhejiang Provincial People’s Hospital (Affiliated People’s Hospital, Hangzhou Medical College), Hangzhou, 310014 Zhejiang China; 3 Emergency and Critical Care Center, Department of Emergency Medicine, Zhejiang Provincial People’s Hospital (Affiliated People’s Hospital, Hangzhou Medical College), Hangzhou, 310014 Zhejiang China; 4 Jinzhou Medical University, Jinzhou, Liaoning Province China; 5 Center for General Practice Medicine, Department of Infectious Diseases, Zhejiang Provincial People’s Hospital, People’s Hospital of Hangzhou Medical College, 158 Shangtang Road, Hangzhou, Zhejiang China; 6 Department of Infectious Diseases, The First Affiliated Hospital of Zhejiang University of Medicine, Hangzhou, Zhejiang China

**Keywords:** Chronic hepatitis B, Liver fibrosis, Serum biomarkers, Machine-learning, Model, Diseases, Risk factors

## Abstract

Early assessment and accurate staging of liver fibrosis may be of great help for clinical diagnosis and treatment in patients with chronic hepatitis B (CHB). We aimed to identify serum markers and construct a machine learning (ML) model to reliably predict the stage of fibrosis in CHB patients. The clinical data of 618 CHB patients between February 2017 and September 2021 from Zhejiang Provincial People's Hospital were retrospectively analyzed, and these data as a training cohort to build the model. Six ML models were constructed based on logistic regression, support vector machine, Bayes, K-nearest neighbor, decision tree (DT) and random forest by using the maximum relevance minimum redundancy (mRMR) and gradient boosting decision tree (GBDT) dimensionality reduction selected features on the training cohort. Then, the resampling method was used to select the optimal ML model. In addition, a total of 571 patients from another hospital were used as an external validation cohort to verify the performance of the model. The DT model constructed based on five serological biomarkers included HBV-DNA, platelet, thrombin time, international normalized ratio and albumin, with the area under curve (AUC) values of the DT model for assessment of liver fibrosis stages (F0-1, F2, F3 and F4) in the training cohort were 0.898, 0.891, 0.907 and 0.944, respectively. The AUC values of the DT model for assessment of liver fibrosis stages (F0-1, F2, F3 and F4) in the external validation cohort were 0.906, 0.876, 0.931 and 0.933, respectively. The simulated risk classification based on the cutoff value showed that the classification performance of the DT model in distinguishing hepatic fibrosis stages can be accurately matched with pathological diagnosis results. ML model of five serum markers allows for accurate diagnosis of hepatic fibrosis stages, and beneficial for the clinical monitoring and treatment of CHB patients.

## Introduction

The estimated prevalence of chronic hepatitis B virus (HBV) infection worldwide in 2016 was 3.5%, with approximately 257 million people living with chronic hepatitis B (CHB)^1^, in whom up to 40% of HBV infections may be progress to decompensated cirrhosis and hepatocellular carcinoma (HCC)^2^. Timely and appropriate antiviral therapy can inhibit viral replication and prevent disease progression, but not all HBV-infected patients require antiviral therapy. The American association for the study of liver diseases (AASLD) recommend that patients with early liver fibrosis (F < 2) need only follow-up observation, while those with significant liver fibrosis (F ≥ 2) definitely require antiviral therapy^3,4^. Therefore, there is a pressing, to search for easily available, relatively inexpensive and reliable biomarkers to assess the stages of liver fibrosis with non-invasive techniques are an urgent.

At present, liver biopsy is still the gold standard when it comes to determine the degree of liver fibrosis. However, biopsy by sampling error, the difference between the observer and the limitation of various potential complications, cause for stable and asymptomatic patients with early liver fibrosis^5–7^. In view of this, non-invasive methods are replacing liver biopsy to evaluate the degree of liver fibrosis. In recent years, the serological detection gets more and more attention, and profit from serum detect is easy to obtain, simple to use, and lower cost, especially in the application of routine screening^8^. Most of these serological detections were developed in viral hepatitis or nonalcoholic fatty liver disease (NAFLD). In general, these tests are assessed by combining markers such as liver injury, complications of poral hypertension, or markers that measure fibrosis generation^9^. However, it should be noted that the sensitivity and specificity of individual serological biomarkers are significantly different, and making them insufficient to accurately evaluate the stages of liver fibrosis in CHB patients. Therefore, there is an urgent and unmet clinical need to find biomarkers that are readily available, relatively inexpensive, and reliable and that can be assessed with less invasive or even noninvasive techniques for assessing the stages of liver fibrosis.

At this stage, the liver fibrosis assessment model combined with multiple serological biomarkers, including Fibrosis 4 score (FIB-4), aspartate aminotransferase to platelet ratio index (APRI) and other classic assessment indicators, is an effective method. However, these methods have reduced the sensitivity and specificity of the test results due to the influence of CHB complications, which can’t accurately reflect the results of liver biopsy^10,11^. Therefore, highly accurate and reliable models are urgently needed in clinical practice to improve decision support for liver fibrosis assessment in CHB patients. In recent years, the application and development of large data and artificial intelligence technology has pioneered the new medical decision-making system, especially the use of ML to improve the reliability and accuracy of diagnostic systems for specific diseases. A preliminary study applying ML to nonalcoholic steatohepatitis (NASH) histology demonstrated the feasibility of this method in assessing liver tissue^12,13^. In this study, we assumed that using new combination of serological biomarkers to construct a predictive model through ML can allows for accurate diagnosis of liver fibrosis in CHB patients. The potential utility of the ML-based risk stratification approach may be demonstrated in comparison with the results of the traditional classic serological model and pathological evaluation.

In conclusion, the purpose of this study was to identify novel predictors of liver fibrosis staging from clinical and serological datasets. Furthermore, a high-flux ML-based predictive model was developed and validated in two independent cohorts based on the identified predictors for the accurate identification of liver fibrosis staging in CHB patients to aid in clinical decision-making.

## Methods

**Figure 1:**
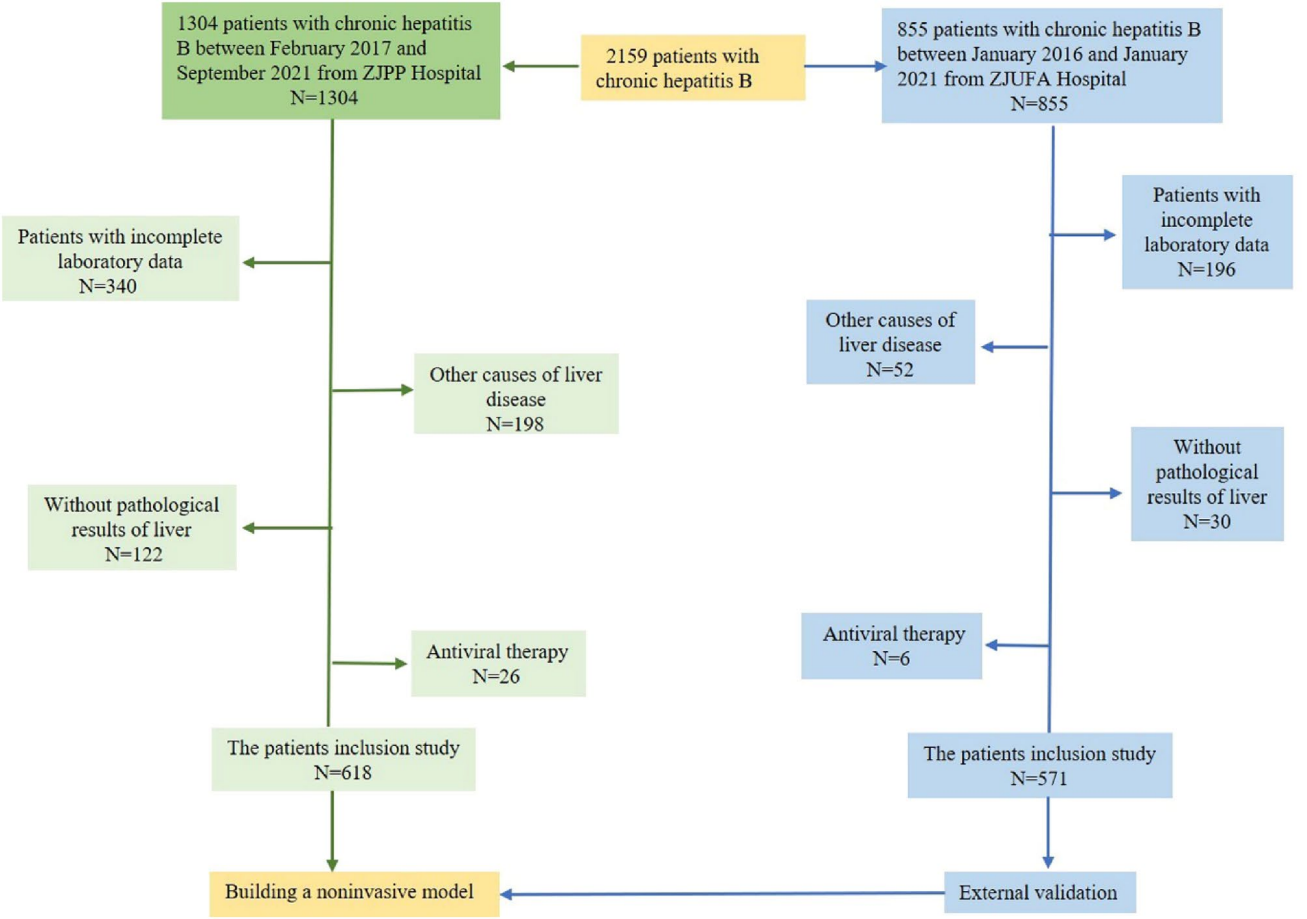
Flowchart of patient recruitment and study design.

This retrospective study was approved by the Ethics Committee of Zhejiang Provincial People’s Hospital. We confirm that all methods were performed in accordance with relevant guidelines and regulations. We retrospectively analyzed the clinical data of 1189 CHB patients from two independent centers, including general information and laboratory data obtained within 1 week prior to liver biopsy. HBV-infected patients over 18 years old with hepatitis B surface antigen (HBsAg) positivity for at least 6 months were included^14^. The exclusion criteria were as follows: ① antiviral therapy; ② incomplete clinical data; ③ other causes of liver disease, such as hepatitis C virus (HCV), alcoholic liver disease, NASH, or autoimmune liver disease. The detailed process of liver biopsy and pathological diagnosis can be found in the Supplementary Material. In this study, we used the dataset from Zhejiang Provincial People’s Hospital as a training cohort to build the model, and we also included 571 HBV-infected patients from the First Affiliated Hospital of Zhejiang University as an independent external validation cohort for the model to further determine the generalizability of the model. We confirm that informed consent was obtained from all subjects and their legal guardian. The flowchart of patient enrollment is shown in Fig. 1.

Statistical analyses of the data were performed with SPSS software (version 25.0, SPSS Inc, IBM,) R 3.5.1 and Python 3.5.6. Quantitative data was tested for normality using the Kolmogorov–Smirnov test and met the normal distribution using the independent sample *t*-test. Non-normally distributed data was analyzed using the Mann–Whitney U test. Counting data was expressed as frequency numbers, and between groups were compared using χ^2^ test. The calculated AUC and 95% confidence interval (CI) were used to evaluate the performance of the ML model for the prediction of grade liver fibrosis. A two-tailed *P* value < 0.05 indicated statistical significance.

## Results

There were significant differences in Alkaline phosphatase, TBIL, DB, AST/ALT and ALT-square among the training cohort and external validation cohort (*P* < 0.05), but there were no significant differences among other characteristics (*P* > 0.05), and detailed feature analysis can be found in Table 1.

**Table 1:** Characteristics of patients in the two cohorts.

Characteristics		All cohort (N = 1189)	Training cohort (N = 618)	Validation cohort (N = 571)	*P*
Clinical features					
Gender(n/%)	male	794(66.67)	413(66.83)	381(66.73)	0.97
	female	395(33.33)	205(33.17)	190(33.27)	
Age		38.06(10.05)	38.56(10.21)	37.51(9.86)	0.073
Quantitative hepatitis B virus					
HBV-DNA		5.53(2.43)	5.52(2.25)	5.56(2.61)	0.778
HBcAb		10.53(2.62)	10.45(2.85)	10.61(2.34)	0.284
Characteristics of coagulation function					
INR		1.06(0.66)	1.08(0.84)	1.04(0.38)	0.242
TT		18.69(3.87)	18.52(5.12)	18.87(1.65)	0.116
Blood routine characteristics					
WBC		5.65(2.45)	5.60(1.76)	5.72(3.02)	0.404
Lymphocyte		1.84(0.67)	1.84(0.72)	1.83(0.62)	0.748
Neutrophils		3.29(1.39)	3.29(1.39)	3.29(1.38)	0.956
RBC		5.37(12.79)	5.39(14.38)	5.35(10.82)	0.955
HB		147.71(21.24)	147.41(24.1)	148.04(17.64)	0.607
Mean erythrocyte volume		90.45(34.18)	91.78(4.69)	89.01(7.09)	0.147
PLT		184.53(78.98)	183.05(58.75)	186.14(96.23)	0.5
Blood biochemical characteristics					
Albumin		44.59(5.10)	44.39(5.58)	44.83(4.53)	0.138
ALT		57.78(82.97)	54.14(81.32)	61.73(84.64)	0.115
AST		39.79(39.87)	38.5(36.59)	41.19(43.12)	0.246
GGT		35.25(41.04)	37.01(46.32)	33.34(34.36)	0.123
Alkaline phosphatase		79.80(30.39)	84.68(27.03)	74.51(32.87)	< 0.001*
TBIL		15.41(7.43)	16.49(7.93)	14.23(6.65)	< 0.001*
DB		3.98(2.54)	3.52(2.39)	4.47(2.6)	< 0.001*
AST/ALT		0.93(0.41)	0.97(0.42)	0.89(0.39)	0.001*
Traditional serological mixed indicators					
APRI		0.63(0.78)	0.62(0.72)	0.64(0.83)	0.596
ALTsquare		6.76(3.47)	6.53(3.39)	7.01(3.55)	0.017*
Fib-4		1.36(1.26)	1.40(1.35)	1.31(1.15)	0.206
AST/PLT		0.25(0.31)	0.25(0.29)	0.26(0.33)	0.596
Fibrosis stages					
F0-1(n/%)		665(55.9)	346(56)	319(55.8)	0.967
F2(n/%)		313(26.3)	162(26.2)	151(26.4)	0.945
F3(n/%)		103(8.7)	54(8.7)	49(8.6)	0.93
F4(n/%)		108(9.1)	56(9.1)	52(9.1)	0.98
Inflammation grades					
G0 (n/%)		12(1)	8(1.3)	4(0.7)	0.306
G1 (n/%)		645(54.2)	345(55.8)	300(52.5)	0.256
G2 (n/%)		405(34.1)	189(30.6)	216(37.8)	0.065
G3 (n/%)		119(10)	70(11.3)	49(8.6)	0.115
G4 (n/%)		8(0.7)	6(1)	2(0.4)	0.191

Data are n, n (%), or mean (SD).

*INR* international normalized ratio, *TT* thrombin time, *ALB* alkaline, *ALT* alanine aminotransferase, *AST* aspartate aminotransferase, *GGT* glutamyl transpeptidase, *TBIL* total bilirubin, *DB* direct bilirub.

* indicates *P* < 0.05.

This study first performed maximum relevance minimum redundancy (mRMR) to exclude redundancy features, and a total of 10 features remained, includes gender, age, HBV-DNA, International NORMALIZED Ratio (INR), thrombin time (TT), Mean erythrocyte volume, albumin, Gamma Glutamyl Transferase (GGT) and Alkaline phosphatase. Second, after dimensionality reduction based on GBDT, 5 features remained. Among them, the weight of HBV-DNA was the highest at 0.261, followed by PLT (0.214), TT (0.183), albumin (0.179) and INR (0.162) (See Figure S2). The variance inflation factor (VIF) is 1.128, 1.01, 1.101, 1.017 and 1.099, which shows no collinearity between the features. The detailed process and results of dimensionality reduction can be found in the Supplementary Materials.

**Figure 2:**
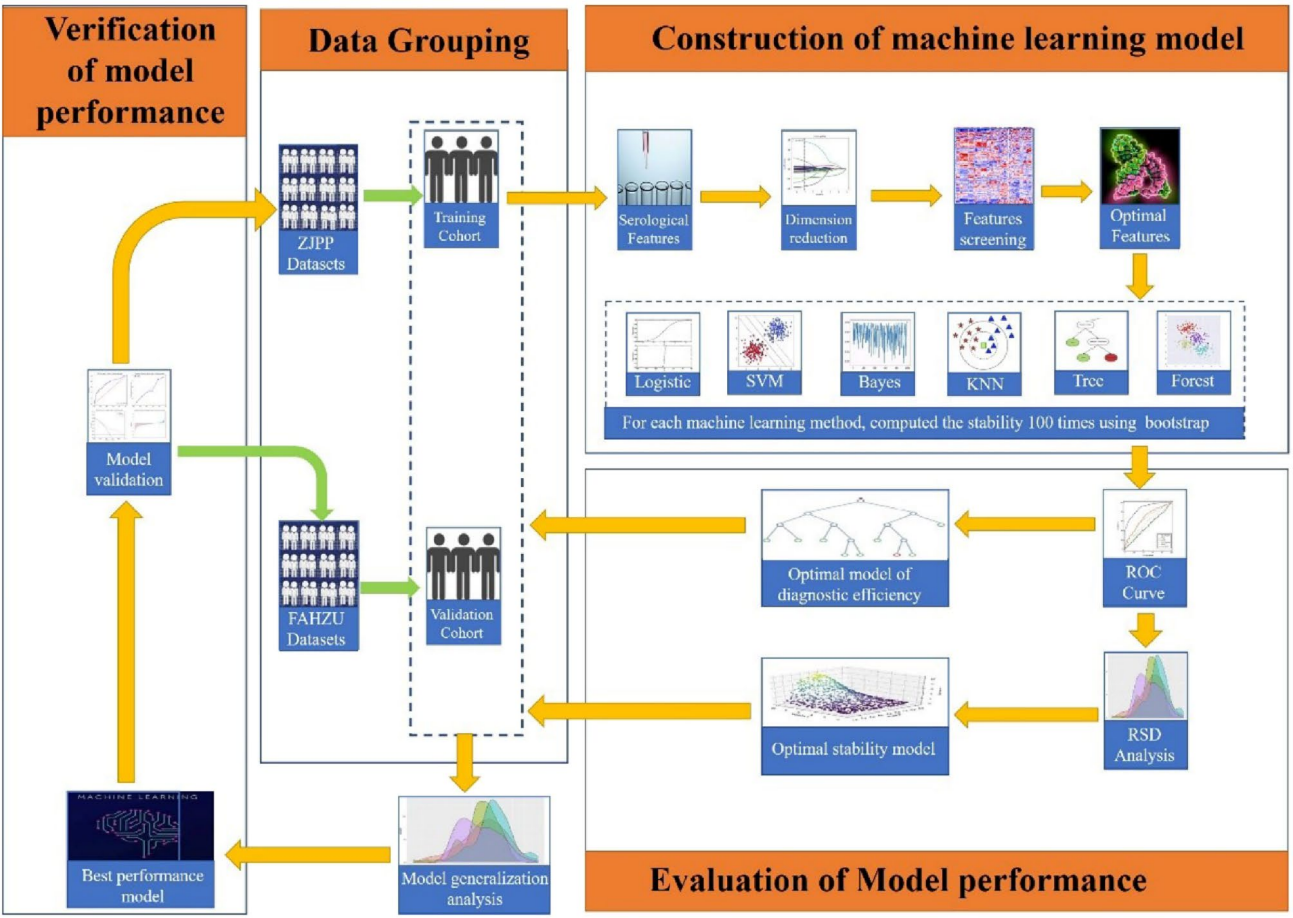
Flowchart of model construction and validation.

**Figure 3:**
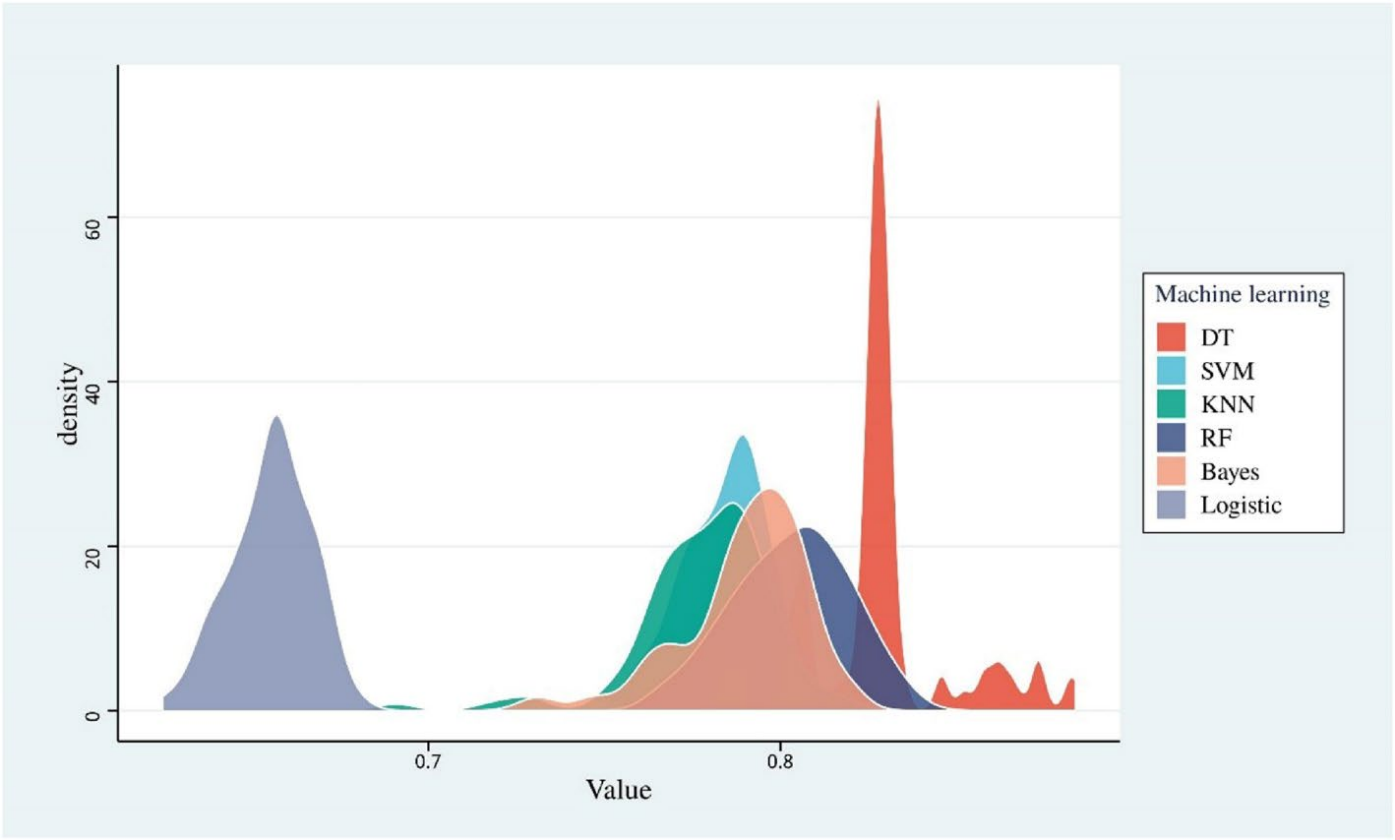
Density chart of AUC values obtained by each machine learning model after 100 resamplings. The SVM model showed the highest density accumulation, and the DT model showed the highest AUC value. *SVM* support vector machine, *KNN* K-nearest neighbor, *DT* decision tree, *RF* random forest, *ML* machine learning.

**Figure 4:**
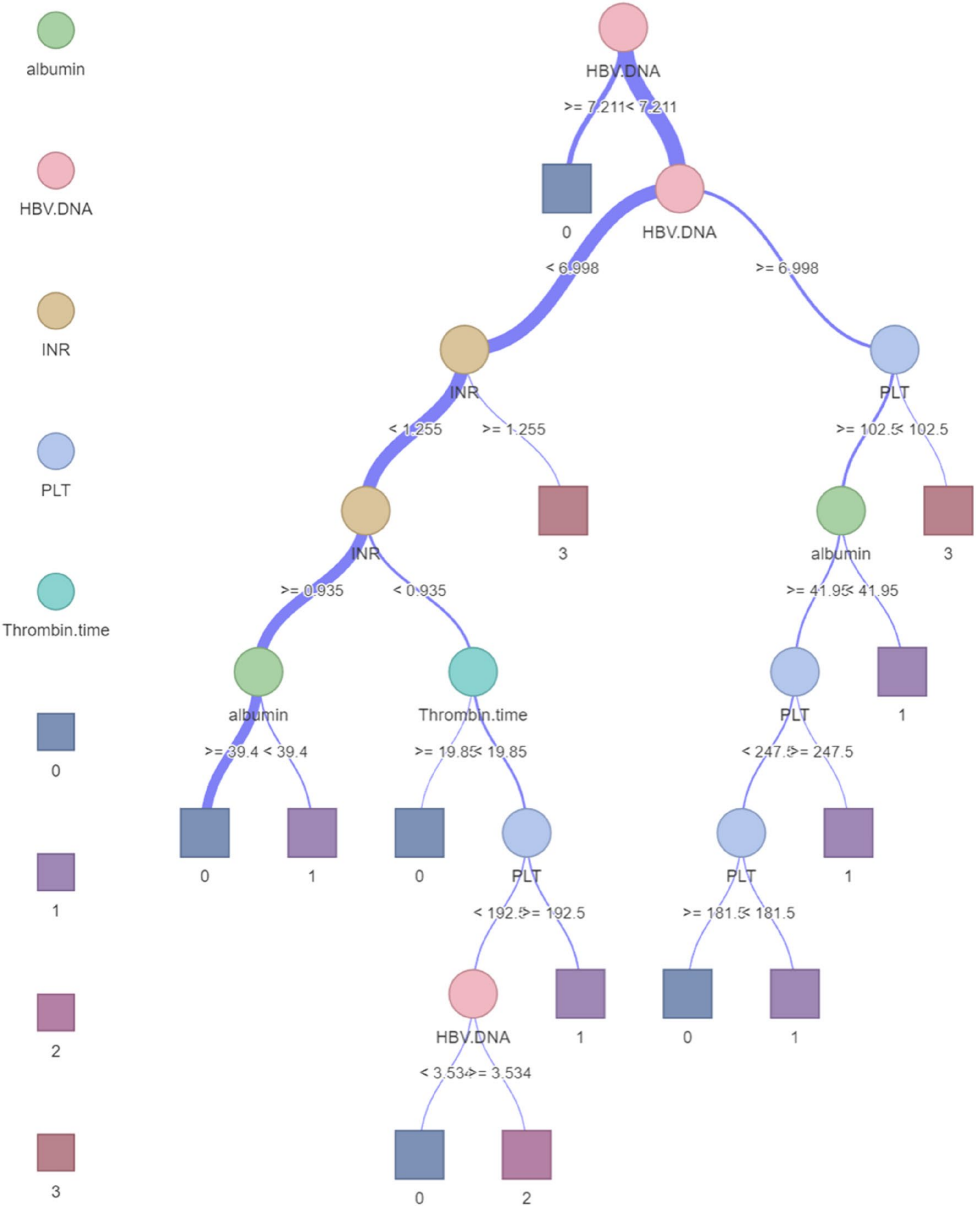
DT model visualization. The square boxes represent clinical outcomes. Darker shades of purple indicate a greater predicted probability value. In this study, the probability value 0 represents the pathological result of F0-1, the probability value 1 represents the pathological result of F2, the probability value 2 represents the pathological result of F3, and the probability value 3 represents a pathological result of F4. *GGT* glutamyl transpeptidase, *INR* international normalized ratio, *PLT* platelet.

The model was constructed using the 5 selected features based on the training dataset (Fig. 2). The model constructed by the SVM ML method showed the best stability, with a mean AUC and RSD of 0.7848 (SD 0.01223) and 1.5583, respectively. The model constructed by the DT ML method showed the best diagnostic performance, with a mean AUC and RSD of 0.8617 (SD 0.02377) and 2.7585, respectively (Fig. 3 and Table S2). To select the best-performing model, the SVM and DT models were applied to the training cohort and the external validation cohort. The RSDs calculated based on the AUC values of the two groups were 1.347 and 0.116, respectively (Table S3). Therefore, the DT model showed the best generalized performance among different independent datasets, and its mode visualization can be seen in Fig. 4.

**Figure 5:**
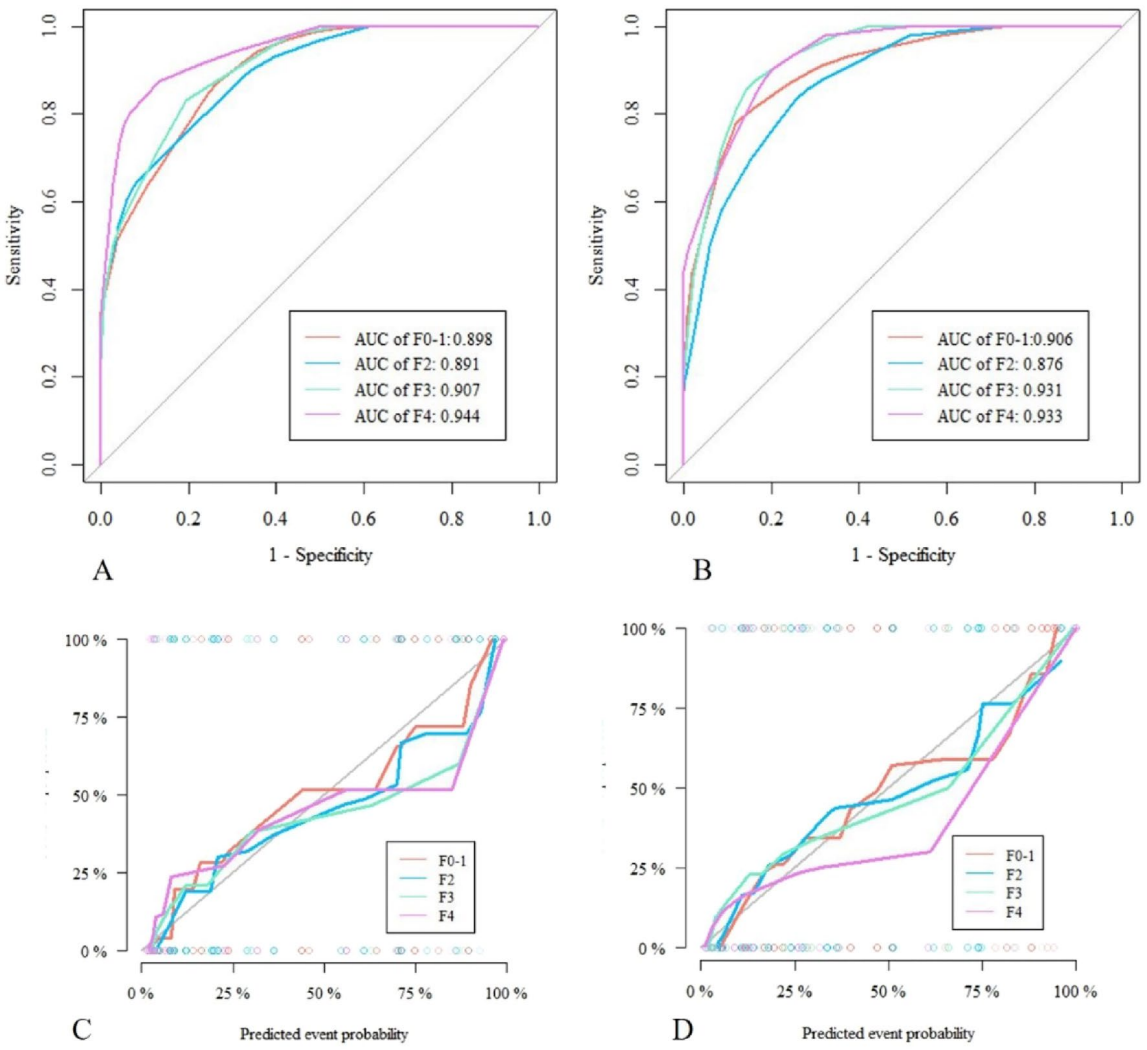
ROC curves of the tree model in the training cohort (**A**) and external validation cohort (**B**). The calibration curves showed the agreement between the predictive performance and the actual results in the training cohort (**C**) and external validation cohort (**D**). The diagonal line represents perfect predictive performance, and the color line represents the model classification performance.

The AUC values of the DT model for assessment of liver fibrosis stages (F0-1, F2, F3 and F4) in the training cohort were 0.898, 0.891, 0.907 and 0.944, respectively. The AUC values of the DT model for assessment of liver fibrosis stages (F0-1, F2, F3 and F4) in the external validation cohort were 0.906, 0.876, 0.931 and 0.933, respectively. The calibration curve of the model showed good agreement with the ideal curve. The Hosmer–Lemeshow test also showed no significant difference between the predicted calibration curve and the ideal curve (P > 0.05) (Fig. 5). In addition, Delong’s test showed that the diagnostic performance of the DT model was significantly different from those of the mixed features in the training cohort and the external validation cohort, highlighting the improvement in the prediction performance of the DT model, as shown in Table 2.

**Table 2:** Diagnostic performance of the ML model and traditional serological model for assessment of hepatic fibrosis stages in the two cohorts.

Cohorts	ML Model	APRI	FIB-4
Cirrhosis(F4) AUC			
Training cohort	0.944*	0.67	0.717
Validation cohort	0.933*	0.69	0.735
Advanced fibrosis(F3) AUC			
Training cohort	0.907*	0.604	0.567
Validation cohort	0.931*	0.63	0.695
Significance fibrosis(F2) AUC			
Training cohort	0.891*	0.515	0.527
Validation cohort	0.876*	0.559	0.551
Early stage of fibrosis(F0-1) AUC			
Training cohort	0.898*	0.602	0.615
Validation cohort	0.906*	0.651	0.681

*AUC* area under the receiver operating characteristic curve, *ML* machine learning, *AUC* of ML model was statistically compared with AUC of FIB-4 and APRI, respectively, in the same fibrosis stage(*P < 0.05).

**Figure 6:**
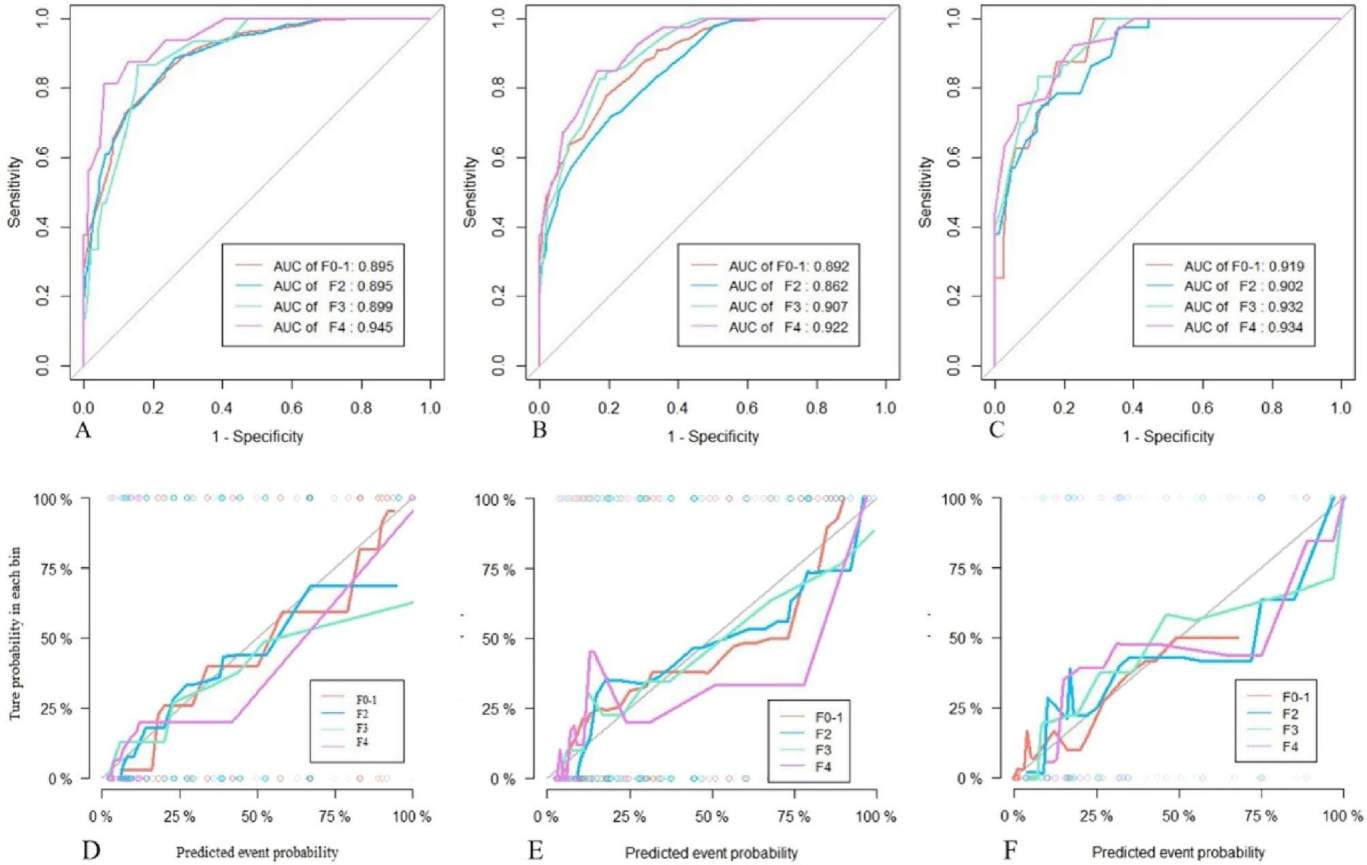
The diagnostic performance of ML model recognizes liver fibrosis grade in mild inflammation (**A**), moderate inflammation (**B**) and severe inflammation (**C**) groups, respectively. The calibration curves showed the agreement between the predictive performance and the actual results in the mild inflammation (**D**), moderate inflammation (**E**) and severe inflammation (**F**) groups. The diagonal line represents perfect predictive performance, and the color line represents the model classification performance.

The DT model calculated the liver fibrosis probability of all patients, and identify early stage of fibrosis(F0-1), significance fibrosis(F2), advanced fibrosis(F3) and Cirrhosis(F4) according to the optimal diagnostic threshold (F0-1 cut-off value: > 0.5, F2 cut-off value: > 0.25714, F3 cut-off value: > 0.05714 and F4 cut-off value: > 0.03846). We observed that there were no significant differences in the number of cases diagnosed by pathology between the four groups, whether in the training or external validation cohorts or even in the entire study cohort (P > 0.05, Table 3), which indicated that the model risk classification effect of this study was good. For all 1189 patients, the Delong’s test showed that the performances of ML model did not significant differences among F0-1, F2, F3 and F4 regarding to different inflammation subgroups (P > 0.05, Table 4 and Fig. 6).

**Table 3:** Clinical classification performance of machine learning model.

Group	ML model results (n, %)		Pathological results (n, %)				Recognition rate (%)	*P* value
			F0-1	F2	F3	F4		
Training cohort (N = 618)	F0-1	302(48.87)	346(56)				87.28	0.581
	F2	116(18.77)		162(26.2)			71.60	
	F3	45(7.28)			54(8.7)		83.33	
	F4	49(7.93)				56(9.1)	87.5	
Validation cohort (N = 571)	F0-1	251(43.96)	319(55.8)				78.68	0.819
	F2	126(22.07)		151(26.4)			83.44	
	F3	46(8.06)			49(8.6)		93.87	
	F4	47(8.23)				52(9.1)	90.38	
Study cohort (N = 1189)	F0-1	553(46.51)	665(55.9)				83.15	0.776
	F2	242(20.35)		313(26.3)			77.32	
	F3	91(7.65)			103(8.7)		88.34	
	F4	96(8.07)				108(9.1)	88.89	

*ML* machine learning.

**Table 4:** Diagnostic performance comparison of ML model for patients with different inflammation levels.

Fibrosis stages	AUC (95%CI)	Sensitivity	Specificity	*P* value
Mild inflammation(G0-1)				
F0-1(n = 503)	0.895(0.869 to 0.917)	0.847	0.766	0.907&
F2(n = 123)	0.895(0.869 to 0.917)	0.886	0.736	0.15&
F3(n = 15)	0.899(0.874 to 0.921)	0.867	0.844	0.821&
F4(n = 16)	0.945(0.925 to 0.961)	0.813	0.942	0.451&
Moderate inflammation(G2)				
F0-1(n = 154)	0.892(0.858 to 0.921)	0.779	0.809	0.516*
F2(n = 153)	0.862(0.824 to 0.894)	0.719	0.794	0.218*
F3(n = 58)	0.907(0.875 to 0.934)	0.828	0.827	0.382*
F4(n = 40)	0.922(0.891 to 0.946)	0.85	0.833	0.644*
Severe inflammation(G3-4)				
F0-1(n = 8)	0.919(0.857 to 0.96)	1	0.714	0.552#
F2(n = 37)	0.902(0.836 to 0.947)	0.973	0.644	0.834#
F3(n = 30)	0.932(0.873 to 0.969)	0.833	0.876	0.395#
F4(n = 52)	0.934(0.876 to 0.971)	0.923	0.773	0.733#

^&^: AUCs of ML model was statistically compared between mild and moderate inflammation groups, respectively, in each fibrosis stage. *: AUCs of ML model was statistically compared between moderate and severe inflammation groups, respectively, in each fibrosis stage. #: AUCs of ML model was statistically compared between severe and mild inflammation groups, respectively, in each fibrosis stage.

## Discussion

In this multicenter study, we designed a prediction model based on ML to accurately assessment liver fibrosis stages of CHB patients. Compared with traditional statistical models such as APRI or FIB-4, and ML model demonstrated significant improvements and was easy to process, which also suggested the great potential of ML in the field of noninvasive liver fibrosis evaluation. In addition, our study results indicated that ML model provided similar diagnostic efficacy with the reference standard liver biopsy, which may provide a reliable theoretical basis for the further development of simple, easy-to-use and accurate tools for the evaluation of liver fibrosis.

In this study, we used ML methods with the hope of more accurately assessing the staging of liver fibrosis, thereby improving the accuracy of the model. The final results revealed that our model showed superior accuracy compared to traditional serological models such as APRI or FIB-4. It is also significantly higher than the diagnostic efficacy of seventeen noninvasive liver fibrosis models in Chinese patients with hepatitis B mentioned in the study of Li et al.^19^. In addition, stratification analysis in inflammation subgroups was performed, and the results did show no significant impact on the performance of ML model. These findings suggest that ML model may overcome the influence of inflammation for cirrhosis evaluation, which is likely to be a potential breakthrough in non-invasive diagnosis. This was helped by a new approach to model building that had the following main advantages. First, we compared the performance of models constructed by several ML methods, and then we focused on and validated the DT model because of its better performance and ease of use. In fact, the DT model has been applied to evaluate hepatitis C liver fibrosis and has shown significant performance^20^. In addition, previous studies mainly used a classification method (logistic regression analysis)^21^, and features were selected through univariate tests (t tests, Welch tests, etc.) in many patients^22,23^. However, this method is often overly optimistic, prone to overfitting, and difficult to reproduce. To overcome these problems, we used integration algorithms, including mRMR and GBDT, to remove redundant features to prevent multicollinearity, and we used only high-scoring variables to construct prediction models to avoid overfitting. Second, our model allows patients to be assessed by a single blood draw without the need for additional modalities. This concept is particularly attractive for routine screening of people at high risk of disease development, such as those with advanced or severe liver fibrosis, in primary care settings. These cases which clinically suspected severe liver fibrosis previously required puncture pathology to be confirmed. However, now only need to routine serological examination to judge the probability of severe liver fibrosis, so invasive puncture examination can be avoided. Therefore, it has obvious advantages in terms of cost and prognosis. In addition, our method can be used to construct a similar model visualization to distinguish early liver fibrosis from significant liver fibrosis, and does not require specially trained clinicians, which is more convenient for clinicians in practice and of great value for clinical promotion.

In this study, we also hoped to improve the diagnostic performance of the model by identifying more specific markers and constructing the model based on the combination of known serologically relevant features. We integrated some of the most routine serological markers, in contrast to Zeng et al., who used laboratory markers such as B2-macroglobulin, haptoglobin and apolipoprotein A1, which are not commonly used in most hospitals^24^. Although these laboratory markers may show higher accuracy than routine serological markers, they are not suitable for practical clinical application. Our results showed of the five conventional serological markers used to construct the ML model, HBV-DNA had the greatest contribution to the model, which is consistent with the recommendation of some guidelines that patients with high HBV-DNA levels should be evaluated for noninvasive liver fibrosis^4,25^. HBV DNA is the marker for viral replication. For chronic HBV infection, the development of the disease is a dynamic process, and the infection status also exists for a long time. For patients with chronic HBV infection in the indeterminate phase, the results of examination alone may not be able to accurately assess the natural history stage, so dynamic follow-up observation is needed. Studies have shown that HBV DNA levels correlated with significant fibrosis in HBeAg(−) CHB patients. HBV DNA level could predict liver fibrosis in HBeAg(−) CHB patients with biopsy indication^26,27^.

In addition, two coagulation factors including INR and TT were integrated into the model, although the two coagulation factors are closely related in clinical practice^28,29^, which was may lead to over fitting of the model and overestimate the role of coagulation factors. However, we calculated the VIF value of relevant factors and did not show collinearity. Therefore, we speculate that the contribution of coagulation factors to the model should not be overestimated.

It is well known that distinguishing F0-1 from F2-4 is more challenging in many studies^30,31^, which is because the heterogeneity of liver fibrosis in patients with F ≥ 2 liver fibrosis is more serious than that in those with F ≥ 3 and 4 liver fibrosis, which generally reduces the accuracy of all classification strategies. In fact, our research results confirm that DT model has the lowest accuracy (AUC of 0.891 in training cohort and AUC of 0.876 in Validation cohort) in identifying patients with liver fibrosis grade F2. However, DT model shows high accuracy and excellent stability for each fibrosis grade in two cohorts, especially in identifying liver cirrhosis (F4), which was shows this model could be used to refine phenotypes in large research studies. Our study result also showed that the highest overall recognition rate for patients with liver cirrhosis (F4) was higher than that for patients with other stages of liver fibrosis when the model was used to classify risk prediction in the two cohorts or the whole cohort. These results suggested that our ML model may be part of a more accurate preclinical detection pathway to assess liver cirrhosis and may be used for the screening and treatment of liver cirrhosis in HBV-infected patients in routine clinical environments, although this needs to be validated in prospective studies.

This study has some limitations. First, this study was a retrospective study, which may lead to the simulation of retrospective statistics depending on too many assumptions. Future research should focus on the development of prediction and classification models based on prospective research, which will allow time evolution information to be used to evaluate, modify and reevaluate prediction models. Second, the model itself needs to be further optimized through better engineering and further development through more comprehensive integration of other clinical data to improve the overall performance of the model and achieve a more accurate noninvasive diagnosis of liver fibrosis staging. Finally, our study did not investigate the performance of ML model for classifying patients with CHB of different ethnic populations, which are also worthy of further studies in the future. Of course, in this study, we still emphasize that as conceptual research, it can still provide a certain basis for the real clinical practice in the future, although this future still needs a long way to go.

In conclusion, this study demonstrated that ML model was more accurate than traditional serological mixed biomarkers in assessing all four liver fibrosis stages in patients with CHB. In addition, the results of this study promote the goal of assessing liver fibrosis in CHB patients and improving the existing prognostic models, thereby facilitating a future prospective study design and evaluation and clinical disease surveillance and treatment. We also hope to further refine and expand this work to clarify the application of this model to a wider range of liver fibrotic diseases.

### Supplementary Information

Supplementary Information.

## Data Availability

The datasets generated and analysed during the current study available from the corresponding author on reasonable request.
