# Supplementary material for: A machine learning-based model analysis for serum markers of liver fibrosis in chronic hepatitis B patients
Source: Sci Rep. 2024 May 27;14:12081. doi: 10.1038/s41598-024-63095-8 (PMC11130122; doi:10.1038/s41598-024-63095-8)
Supplement: Supplementary file 1 — Supplementary Information. [file 41598_2024_63095_MOESM1_ESM.docx]

**A machine learning-based model analysis for serum markers of liver fibrosis in chronic hepatitis B patients**

Congjie Zhang^1#^, Zhenyu Shu^2#^, Shanshan Chen^3#^, Jiaxuan Peng^4^, Yueyue Zhao^5^, Xuan Dai^5^, Jie Li^5^, Xuehan Zou^5^, Jianhua Hu^6^, Haijun Huang^5*^

**Supplementary Information**

1. **Liver puncture biopsy**

Ultrasound-guided liver puncture biopsy is performed as follows: the patient lies flat on his back with his right hand elevated above his head, and ultrasound is used to locate the liver puncture site (usually the right liver is chosen). Each liver tissue specimen was required to be greater than 15mrn in length and contain 6 manifold areas.

**2.Pathological diagnosis of liver fibrosis**

The liver histopathology results of the patients with chronic hepatitis B enrolled in this study were re-read by three experienced pathologists to determine the degree of liver fibrosis. All specialists were evaluated without knowledge of patient characteristics and re-evaluated if results were inconsistent. Pathological diagnostic criteria: fibrosis staging of liver tissue according to the METAVIR scoring system: F0 no fibrosis; F1 fibrous enlargement of the confluent area but no fibrous septum formation; F2 fibrous enlargement of the confluent area with little fibrous septum formation; F3 most fibrous septum formation but no sclerotic nodules; F4 cirrhosis.

3.**Acquisition of laboratory indicators**

All laboratory indexes were obtained by the laboratory department of the medical center and Zhejiang first medical center or the State Key Laboratory for diagnosis and treatment of infectious diseases. The details of instrument were found from Table S1. In addition, APRI and FIB-4 serological mixed model formulas can be follows:

$$APRI=\frac{AST(IU/L)/ULN((upper limit of mormal))}{PLT({10}^{9}/L)}\times100$$

$$FIB-4=\frac{\mathrm{Age}\left( \mathrm{year} \right)\times AST\left( \mathrm{IU}/L \right)}{\mathrm{PLT}\left( {10}^{9}/L \right)\times\surd\mathrm{ALT}\left( \mathrm{IU}/L \right)}\times100\left[ 1-2 \right]$$

4. **Details of Dimension reduction**

The minimum redundancy maximum relevance (mRMR) algorithm was used to extract robust features from the training set. The aim of the minimum redundancy process ensured that the selected features had minimal redundancy among the other features. At the same time, maximum relevance procedure was to select features having the maximum correlation with the actual grading of liver fibrosis，and we selected features with correlation coefficients greater than 0.8 and 0.1 as high correlation and low redundancy features, respectively. Then, the mRMR method was used to obtain an optimal feature set with a high correlation and low redundancy.

Finally, the gradient boosting decision tree (GBDT) algorithm was used to reduce the dimension of the remaining features. GBDT is an algorithm that classifies or regresses data by the linear combination of basis functions and reduces the residual generated in the training process. In this study, five features were obtained from the GBDT procedure. Figure S1 shows the dimension reduction process of mRMR, Figure S2 shows the weight values of the remaining features after the dimensionality reduction of GBDT. **
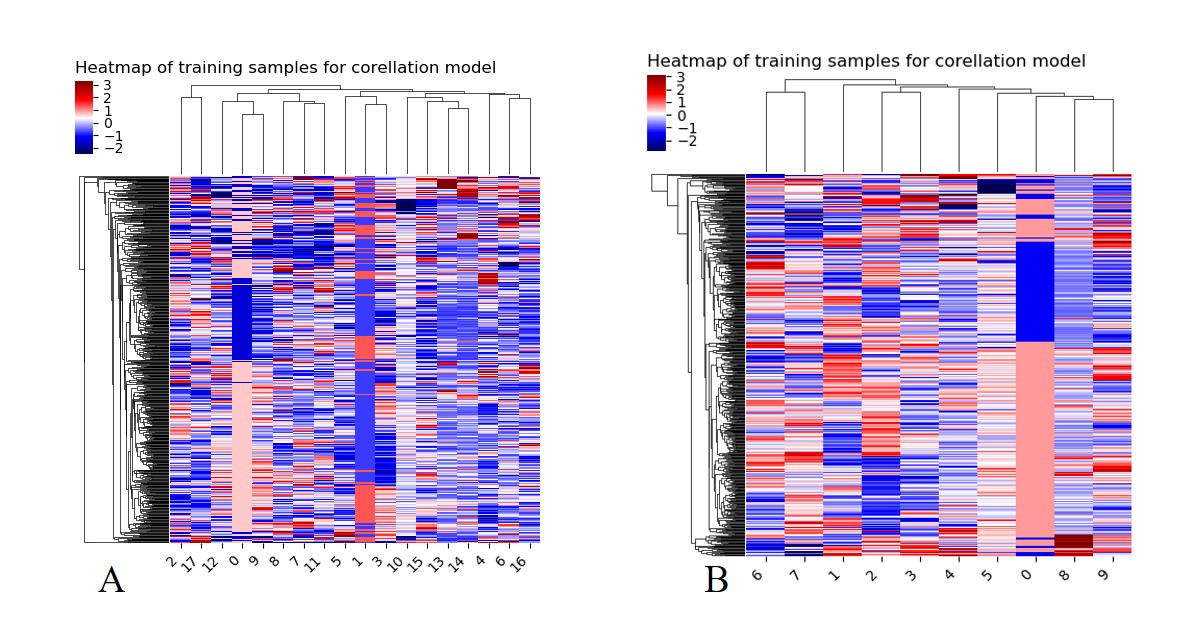
**

**Figure S1.** Process of heatmap for dimension reduction, A and B show the heatmap of the remaining features after dimensionality reduction using mRMR, respectively. Abscissa represents feature ordering, ordinate represents case sequencing, and color represents feature value size.

**
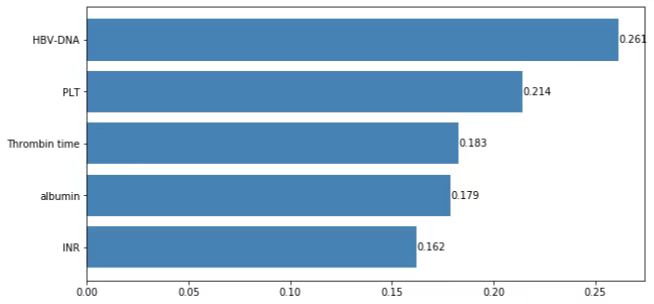
**

**Figure S2.** The ranking chart of the weight values of the remaining 5 features after GBDT dimensionality reduction.

**5. Details of Machine Learning**

In our study, ten-fold cross-validation involved stratifying and dividing the training data into 10 folds of equal size: 8 folds (80%) for training, 1 fold (10%) for tuning model parameters, and 1 fold (10%) for testing. Stratification was used to ensure a similar distribution of events across the 10 folds. This process was repeated for 10 iterations, always using a different data fold for model training, tuning, and testing. We used 6 machine-learning classifiers (Logistic Regression, Bayes, Decision tree, K-Nearest neighbors, Random forest and Support vector machines), and the machine learning scores were concatenated from all 10 testing data folds to allow assessment of model performance over the entire dataset. Each of our 5 machine learning models was cross-validated separately using the same set of iterations and same folds of patient data. All classifiers were implemented using R package caret [3], which provides a nice interface to access many machine-learning algorithms in R. Furthermore, it also provides a user-friendly framework for training different machine-learning models. We used parameter configurations of machine learning that were previously defined by Fernandez-Delgado et al [4], and we have listed the classification methods along with their parameters and corresponding R packages. At last, we focused on and validated the Decision tree model because of its better performance and ease-to-use. Details of the results are shown in Table S2 and S3.

**Logistic Regression (LR)**

R package “rms” with a function lrm was defined to generate a textual description of a logistic regression model, including AUC values and weights for each feature was used for implementation of LR classifier.

**Decision tree (DT)**

A C5.0 decision tree based classification method was used in the analysis. C5.0 function of the “C50” package was used for creating classification trees with default parameter tuning under caret interface.

**Bayesian (BY)**

R package “klaR” with default caret parameter tuning was used for the implementation of Naïve Bayes classifier.

**Random forest (RF)**

Random forest provides an improvement to bagging with a modification step of random sampling of predictors. R package “randomForest” with caret interface was used for the implementation. Parameter ntree was set to 500 and mtry was varied with values 2:3:29 (2 to 29 with an increment step of 3).

**Support vector machine (SVM)**

SVM, with Gaussian kernel function was implemented using a caret interface and R package kernlab. Cost parameter C was varied with values {2^-2^, 2^-1^ ,1, 2^1^, 2^2^} and the parameter kernel spread was varied with values in {10^-2^, 10^-1^, 1, 10^1^ ,10^2^}.

**K-Nearest neighbor (KNN)**

K-nearest neighbor was implemented using the “knn” R package and caret interface. 10 different values of number of neighbors 5:2:23 (5 to 23 with an increment step of 2) were used.

**References**

1. Wai CT, Greenson JK, Fontana RJ, et al. A simple noninvasive index can predict both significant fibrosis and cirrhosis in patients with chronic hepatitis C. Hepatology. 2003, 38(2):518-26.
2. Sterling RK, Lissen E, Clumeck N, et al. APRICOT Clinical Investigators. Development of a simple noninvasive index to predict significant fibrosis in patients with HIV/HCV coinfection. Hepatology. 2006, 43(6):1317-25.
3. Kuhn M. Building predictive models in R using the caret package. J Stat Softw. 2008, 28(5):1–26.
4. Fernández-Delgado, Cernadas, Barro, et al. Do we need hundreds of classifiers to solve real world classification problems? J. Mach. Learn. Res. 2014, 15:3133–3181.

**Table S1. Laboratory index testing instrument**

| Features | Instrument manufacturing company | Instrument model |
| --- | --- | --- |
| HBV-DNA level | | |
| ZJPP hospital | Applied Biosystems | ABI7300 |
| ZJUFA hospital | Shanghai Zhijiang Biotechnology Co., Ltd | SLAN-96S |
| Coagulation function indexes | | |
| ZJPP hospital | Sysmex Corporation | Sysmex cs-5100 |
| ZJUFA hospital | Sysmex Corporation | Sysmex cs-5100 |
| Blood routine indexes | | |
| ZJPP hospital | Sysmex Corporation | Sysmex XN-9000 |
| ZJUFA hospital | Sysmex Corporation | Sysmex XE-5000 |
| Blood biochemical indexes | | |
| ZJPP hospital | Beckman Coulter, Inc | AU5800 |
| ZJUFA hospital | HITACHI Corporation | HITACHI 7600-210 |

**Table S2. Predictive performances of different machine learning methods**

| Model | Mean value | Standard deviation | RSD(%) |  |
| --- | --- | --- | --- | --- |
| LR | 0.6553 | 0.01126 | 1.7183 |  |
| SVM | 0.7848 | 0.01223 | 1.5583 |  |
| Bayes | 0.7898 | 0.01788 | 2.2639 |  |
| KNN | 0.7784 | 0.01922 | 2.4692 |  |
| DT | 0.8617 | 0.02377 | 2.7585 |  |
| RF | 0.8030 | 0.01622 | 2.0199 |  |

Note: AUC: area under the curve; SD: Standard Deviation; LR: logistic regression, SVM: support vector machine, KNN: K-nearest neighbor, DT: decision tree, RF: random fore

**Table S3. ROC curve analysis of the SVM and DT models in different cohorts**

| Fibrosis stages | Training Cohort | | | Validation Cohort | | | RSD(%) |
| --- | --- | --- | --- | --- | --- | --- | --- |
|  | AUC (95%CI) | Sensitivity | Specificity | AUC (95%CI) | Sensitivity | Specificity |  |
| **SVM Model** | | | | | | | 1.347 |
| F0-1 | 0.761(0.725 to 0.794) | 0.647 | 0.775 | 0.784(0.748 to 0.818) | 0.687 | 0.774 |  |
| F2 | 0.705(0.667 to 0.74) | 0.605 | 0.73 | 0.74(0.702 to 0.775) | 0.755 | 0.636 |  |
| F3 | 0.811(0.777 to 0.841) | 0.704 | 0.793 | 0.779(0.743 to 0.813) | 0.898 | 0.59 |  |
| F4 | 0.839(0.808 to 0.867) | 0.768 | 0.76 | 0.871(0.84 to 0.897) | 0.827 | 0.813 |  |
| **DT Model** | | | | | | | 0.116 |
| F0-1 | 0.898(0.871 to 0.921) | 0.873 | 0.735 | 0.906(0.879 to 0.928) | 0.787 | 0.873 |  |
| F2 | 0.891(0.815 to 0.873) | 0.716 | 0.849 | 0.876(0.846 to 0.902) | 0.834 | 0.743 |  |
| F3 | 0.907(0.849 to 0.902) | 0.833 | 0.805 | 0.931(0.907 to 0.951) | 0.878, | 0.837 |  |
| F4 | 0.944(0.923 to 0.61) | 0.875 | 0.868 | 0.933(0.909 to 0.952) | 0.904 | 0.798 |  |

*Abbreviations*: AUC: area under the curve; SD: standard deviation; SVM: support vector machine; DT: decision tree. RSD: relative standard deviation.
